# Olfactory Mucosa Mesenchymal Stem Cells Alleviate Cerebral Ischemia/Reperfusion Injury *Via* Golgi Apparatus Secretory Pathway Ca^2+^ -ATPase Isoform1

**DOI:** 10.3389/fcell.2020.586541

**Published:** 2020-10-30

**Authors:** Jialin He, Jianyang Liu, Yan Huang, Yi Zhuo, Wei Chen, Da Duan, Xiangqi Tang, Ming Lu, Zhiping Hu

**Affiliations:** ^1^Department of Neurology, The Second Xiangya Hospital, Central South University, Changsha, China; ^2^Key Laboratory of Protein Chemistry and Developmental Biology of Ministry of Education, College of Life Sciences, Hunan Normal University, Changsha, China; ^3^Hunan Provincial Key Laboratory of Neurorestoratology, Second Affiliated Hospital of Hunan Normal University, Changsha, China; ^4^Department of Neurosurgery, Second Affiliated Hospital of Hunan Normal University, Changsha, China

**Keywords:** ischemic stroke, SPCA1, olfactory mucosa mesenchymal stem cells, Golgi apparatus stress, Ca^2+^ overload

## Abstract

Olfactory mucosa mesenchymal stem cells (OM-MSCs) have exhibited their effectiveness in central nervous system diseases and provided an appealing candidate for the treatment of ischemic stroke. Previous evidence have shown that Golgi apparatus (GA) secretory pathway Ca^2+^-ATPase isoform1 (SPCA1) was a potential therapeutic target for ischemic stroke. In this study, we explored the neuroprotective mechanism of OM-MSCs and its effect on the expression and function of SPCA1 during cerebral ischemia/reperfusion. Based on *in vitro* and *in vivo* experiments, we discovered that OM-MSCs attenuated apoptosis and oxidative stress in ischemic stroke models, reduced the cerebral infarction volume, and improved the neurologic deficits of rats. OM-MSCs also upregulated SPCA1 expression and alleviated Ca^2+^ overload and decreased the edema and dissolution of the GA in neurons. Moreover, we discovered that SPCA1 depletion in oxygen and glucose deprivation/reoxygenation (OGD/R)-treated N2a cells mitigated the protective effects of OM-MSCs. Altogether, OM-MSCs exerted neuroprotective effects in ischemic stroke probably *via* modulating SPCA1 and reducing the edema and dissolution of the GA in neurons.

## Introduction

Stroke is a leading cause of death and disability worldwide, in which ischemic stroke accounts for approximately 71% of all stroke types. In 2017, the global incidence of ischemic stroke events was about 7.7 million, with 2.7 million deaths (GBD 2017 Disease and Injury Incidence and Prevalence Collaborators, 2018; Campbell et al., 2019). However, the available therapy options regarding ischemic stroke have limited effects (Huang et al., 2018). Current treatments for acute ischemic stroke are based on reperfusion through thrombolysis or endovascular therapy, but both therapies are limited by the therapeutic time window: thrombolysis by the recombinant tissue plasminogen activator (rtPA) is required within 4.5 h of onset; endovascular therapy, 6 h. Although endovascular therapy can be extended to 24 h if the patient meets the inclusion criteria (Nogueira et al., 2018), only a few patients can actually benefit from it on account of the strict inclusion criteria and the narrow therapeutic time windows. Therefore, many researchers are actively looking for other effective treatments for ischemic stroke, such as cell therapy.

Previous preclinical researches have shown that stem cell transplantation could lead to functional improvement of ischemic stroke animal models (Wei et al., 2017; Boncoraglio et al., 2019). The stem cells involved in these studies included neural stem cells (NSCs; Boese et al., 2018), mesenchymal stem cells (MSCs; Kranz et al., 2010; Oshita et al., 2020), induced pluripotent stem cells (iPSCs; Gervois et al., 2016), and so on. Due to the diversity of access sources, multiple differentiation potential, and the plasticity of function, MSCs have become an appealing stem cell candidate for the treatment of ischemic stroke (Stonesifer et al., 2017; Wang et al., 2018).

Olfactory mucosa mesenchymal stem cells (OM-MSCs), first identified by Tome et al. (2009), are a type of Nestin-positive MSCs that reside in the lamina propria of the olfactory mucosa, having the potential to differentiate into osteocytes, adipocytes, smooth muscle cells, and neurons (Delorme et al., 2010). OM-MSCs are easily accessible, exhibit an extensive proliferation rate, and eliminate ethical concerns compared with the other stem cell types (Lindsay et al., 2020). Moreover, OM-MSCs promoted central nervous system myelination *in vitro* by secretion of the chemokine CXCL12, which was not related to bone marrow mesenchymal stem cells (BM-MSCs; Lindsay and Barnett, 2017). Another research demonstrated that OM-MSCs had a stronger secretion of immunosuppressive cytokines than adipose-derived mesenchymal stem cells (AD-MSCs; Jafari et al., 2020). The aforementioned advantages supported that OM-MSCs may be an appealing candidate of cell therapies for the treatment of human diseases.

Accumulating evidence showed that OM-MSCs exhibited effectiveness and potential in central nervous system diseases, including spinal cord injury, early-onset sensorineural hearing loss, and hippocampal lesions (Lindsay and Barnett, 2017; Lindsay et al., 2017; Zhuo et al., 2017). Huang et al. have concluded that OM-MSCs could inhibit pyroptotic and apoptotic death of microglial cells during ischemia/reperfusion (Huang et al., 2020). However, the impact of OM-MSCs on neuronal injury in ischemic stroke remains unclear.

At present, the inhibition of reperfusion injury is the key to the treatment of ischemic stroke. Intracellular oxidative stress and Ca^2+^ overload are the pivotal pathological processes of cerebral ischemia/reperfusion injury (IRI), leading to irreversible neuronal damage. Apart from mitochondria and lysosomes, Golgi apparatus (GA) also participates in the process of oxidative stress. Jiang et al. (2011) have presented the concept of “GA stress,” which consisted of the activity of Ca^2+^-ATPase in GA; the morphology and membrane surface components of the GA would change correspondingly under oxidative stress.

There are Ca^2+^ release channels and Ca^2+^ uptake mechanisms in the GA (Li et al., 2013). The Golgi-resident secretory pathway Ca^2+^-ATPase (SPCA), which is highly expressed in the brain, is mainly responsible for transporting Ca^2+^ from the cytoplasm to the Golgi lumen and is involved in cytosolic and intra-Golgi Ca^2+^ homeostasis (He and Hu, 2012; Li et al., 2013). SPCA comprises secretory pathway Ca^2+^-ATPase isoform1 (SPCA1) and SPCA2, encoded by ATP2C1 and ATP2C2, respectively, (Hu et al., 2000; Xiang et al., 2005). SPCA1 is well understood, while the function of SPCA2 is rarely studied.

Previous studies have found that oxidative stress may exert the ability to downregulate the expression of SPCA1 in ischemia/reperfusion rats (Pavlíková et al., 2009; Li et al., 2015; Fan et al., 2016). Besides, SPCA1 was found to be able to protect cells from oxidative stress by interacting with the *HSP60* gene (Uccelletti et al., 2005), while the inhibition of SPCA1 function could lead to apoptosis in N2a cells (Sepulveda et al., 2009) and mice (Okunade et al., 2007). Furthermore, the inactivation of SPCA1 could induce the alteration of the mitochondrial structure and metabolism, which would make the mitochondria more sensitive to oxidative stress (He and Hu, 2012). Based on existing evidences, improving the expression and function of SPCA1 was expected to be a therapeutic target for cerebral IRI.

In the present study, we explored the neuroprotective mechanism of OM-MSCs during cerebral ischemia/reperfusion and its effect on the expression as well as function of SPCA1 and further investigated the role of SPCA1 knockdown in the neuroprotective effect of OM-MSCs on cerebral IRI.

## Materials and Methods

### Ethic Statement

Olfactory mucosa mesenchymal stem cells were obtained from two healthy male volunteers for scientific purposes (21 and 28 years old, respectively) at the Second Affiliated Hospital of Hunan Normal University. Human nasal mucosa biopsies were performed by otolaryngology endoscopy operation at the Department of Otolaryngologic Surgery, the second affiliated hospital of Hunan Normal University (Changsha, China). Written informed consent was given by each individual participating in the study before the operation, in accordance with the Helsinki Convention (1964). The investigators and all procedures were approved by the ethics committee of Hunan Normal University (ethical approval document no. 2018–30).

### Isolation and Characterization of OM-MSCs

The isolation and culture of OM-MSCs were carried out using a protocol from a previous study (Ge et al., 2016). Briefly, olfactory tissue samples were obtained from the root of the medial aspect of the middle turbinate undergoing endoscopic nasal surgery, washed three times at room temperature with penicillin streptomycin solution (Invitrogen, Carlsbad, CA, United States), and then cultured in Dulbecco’s modified Eagle’s medium/nutrient mixture F12 (DMEM/F12; Invitrogen, United States) containing 10% fetal bovine serum (FBS; Gibco, Australia) and incubated at 37°C in 5% CO_2_. OM-MSCs at passages 3 and 4 were used for further experiments. Cell surface markers were used to characterize OM-MSCs by flow cytometric analysis.

### Oxygen and Glucose Deprivation/Reoxygenation

Mouse N2a cells were purchased from the Cell Storage Center of the Chinese Academy of Sciences (Shanghai, China). N2a cells were cultured in DMEM (Sigma, United States) supplemented with 10% FBS (Gibco, Australia) at 37°C in 5% CO_2_.

To achieve ischemic-like conditions *in vitro*, the oxygen and glucose deprivation/reoxygenation (OGD/R) model was performed as previously described (Huang and Hu, 2018). Simply, the N2a cells were placed in a modular incubator chamber (Billups Rothenberg, Inc., Del Mar, CA, United States), which kept the pO_2_ value consistently below 0.5%. The culture medium was replaced with deoxygenated glucose-free Hanks’ Balanced Salt Solution (Sigma). The cells were maintained in the hypoxic and glucose-free chamber for 4 h. After OGD, the N2a cells were quickly maintained in DMEM without FBS and incubated under normoxic conditions for 0, 4, 12, and 24 h. N2a cells cultured with DMEM containing 10% FBS in normoxia (5% CO_2_, 37°C) were used as normal controls.

### Co-culture of N2a Cells and OM-MSCs

The co-culture system was set up as previously described (Wei et al., 2019). In brief, the N2a cells (1 × 10^5^) grown in six-well plates were subjected to stress by the OGD method, as described above. At the same time as reoxygenation begins, the N2a cells were rescued by plating 1 × 10^5^ OM-MSCs (OM-MSCs : N2a = 1:1) on the Transwell membrane inserts (pore size, 0.4 μm; polycarbonate membrane, Corning, United States) and incubating for 24 h. During reperfusion, DMEM without FBS conditioning media were used.

### Animals

All animal procedures were approved by the Laboratory Animal Ethics Committee of the Second Affiliated Hospital of Hunan Normal University (ethical approval document no. 2020–164). All experimental procedures were performed in accordance with the Guide for the Care and Use of Experimental Animals. Male Sprague–Dawley rats weighing 250–300 *g* were kept under controlled housing conditions with a 12-h light/dark cycle with food and water *ad libitum*.

### Rats Reversible Middle Cerebral Artery Occlusion Model and OM-MSC Transplantation

The right reversible middle cerebral artery occlusion (MCAO) model was performed as previously described (Longa et al., 1989). Briefly, rats were fasted for 12 h before surgery with water accessible. Rats were initially anesthetized with 3.5% isoflurane and maintained with 1.0–2.0% isoflurane in 2:1 N_2_O/O_2_ using a face mask. The right common carotid artery (CCA), internal carotid artery (ICA), and external carotid artery (ECA) were separated, and an incision was made on the carotid artery using ophthalmic scissors. A surgical filament (0.26-mm diameter; Beijing Cinontech Co. Ltd.) was inserted into the ICA from the incision of CCA, with the length of the line being 18–20 mm. Resistance implied that the line had reached the beginning of the right middle cerebral artery (MCA), thus blocking the blood flow of the vessel. The filament was withdrawn after 120 min, after which the skin wound was sutured. The body temperature of the rats was maintained at 37 ± 0.5°C during the whole procedure. For analgesia, post-surgery rats were given a subcutaneous injection of morphine (2.5 mg/kg) every 4 h for 1 day following MCAO.

In total, 72 adult male Sprague–Dawley rats were randomly divided into three groups: sham operation group (sham), MCAO group (ischemia/reperfusion, I/R), and MCAO + OM-MSC group (transplantation; *n* = 24 animals per group). In the transplantation group, the rats received tail vein injection of 5.0 × 10^6^ OM-MSCs dissolved in 1 ml saline at 24 h after MCAO model induction, while the rats received tail vein injection of 1 ml saline in the I/R group. Rats in each group were sacrificed after anesthesia for experiment on day 7 after reperfusion. All experimental procedures were performed by investigators blinded to group allocation.

### Inclusion and Exclusion Criteria

The inclusion and exclusion criteria of the I/R and transplantation groups were based on the Zea-Longa score when the rats were awake after operation (Longa et al., 1989). The following are the scoring criteria: 0 point = there is no any neurological symptom; 1 point = the left forelimb of the rats cannot entirely stretch; 2 points = Sprague–Dawley (SD) rats rotate to the ischemic side while walking, moderate neurological deficit; 3 points = SD rats dump to the ischemic side when standing; and 4 points = SD rats cannot walk on their own and lose consciousness. Specifically, SD rats with a score of 1–3 were used in the subsequent experiment, while SD rats that died, or with a score of 0 or 4, were dropped. To compensate for dropouts, three additional animals were enrolled to the study population, resulting in an overall study population of 75 rats.

### CCK-8 Assay

Cell viability in each group was measured by using the Cell Counting Kit-8 (CCK-8; Dojindo Molecular Technologies, Dojindo, Japan) according to the manufacturer’s protocol.

### LDH Measurement

Immediately following OGD/R, the culture supernatants were collected and, subsequently, the level of lactate dehydrogenase (LDH) was detected using the LDH Cytotoxicity Assay Kit (Nanjing Jiancheng Bioengineering Institute, Jiangsu, China) according to the manufacturer’s protocol.

### Apoptosis Measurement

Apoptosis of N2a cells and the ipsilateral cortex of SD rats were detected *via* annexin V–fluorescein isothiocyanate (FITC) and propidium iodide (PI) double staining using a FITC Annexin V Apoptosis Detection Kit I (KeyGen Biotech, Jiangsu, China) according to the manufacturer’s instructions. The fluorescence was measured by flow cytometry (Beckman, United States).

### TUNEL and NeuN Double Immunostaining

Apoptosis of neurons in the ipsilateral cortex of SD rats was evaluated by terminal-deoxynucleotidyl transferase-mediated nick-end labeling (TUNEL) and NeuN double immunostaining according to the manufacturer’s protocol. The brain sections from each group were incubated with TUNEL reaction mixture (Beyotime, Shanghai, China) for 1 h at room temperature and then stained with anti-NeuN (ab177487, Abcam, Cambridge, United Kingdom) and DAPI (Wellbio, China). The slides were observed using a fluorescence microscope (Motic, China).

### ROS Measurement

Intracellular reactive oxygen species (ROS) was detected using an oxidation-sensitive fluorescent probe (2′,7′-dichlorodihydrofluorescein diacetate, DCFH-DA). Following OGD/R, reactive oxygen species detection was performed using a fluorescent probe DCFH-DA kit (Beyotime). The cells were washed twice with phosphate-buffered saline (PBS) and subsequently incubated with 10 μmol/L DCFH-DA at 37°C for 20 min. After washing three times, the fluorescence was measured by flow cytometry (Beckman).

### LPO and T-SOD Measurements

The ipsilateral cortex of the SD rats from each group was used for total superoxide dismutase (T-SOD) and lipid peroxidation (LPO) measurements. The levels of LPO and T-SOD were detected using lipid peroxidation and T-SOD assay kits (Nanjing Jiancheng Bioengineering Institute) according to the manufacturer’s instructions.

### Quantitative Real-Time PCR

Total RNA was isolated from N2a cells and ipsilateral brain samples of SD rats using the TRIzol^TM^ Reagent (Thermo Fisher Scientific, United States). Reverse transcription was performed using a reverse transcription kit (Beijing ComWin Biotech Co., Ltd., China). Quantitative PCR (qPCR) was performed using UltraSYBR Mixture (Beijing ComWin Biotech Co., Ltd.). The following qPCR primer sequences were used for messenger RNA (mRNA) quantification: 5′-AGAACTTATTGCCTCCGTCC-3′and 5′-ATCTTTTGAAATCGTGCAACCTG-3′for mouse SPCA1, 5′-ACATCCGTAAAGACCTCTATGCC-3′and 5′-TAC TCCTGCTTGCTGATCCAC-3′for mouse β-actin.

### Western Blot Assay

N2a cells and the ipsilateral cortex of SD rats were processed for Western blot as described (Fan et al., 2016). Immunoblot analyses were performed using the following primary antibodies: anti-SPCA1 (ab126171, Abcam) and anti-β-actin (60008-1-Ig, Proteintech, United States). The anti-rabbit IgG and anti-mouse IgG secondary antibodies were obtained from Proteintech. The proteins were visualized using an enhanced chemiluminescent (ECL) detection kit (Advansta Inc., United States).

### Intracellular Ca^2+^ Measurement

For N2a cells, Golgi vesicles were isolated by a GA protein extraction kit (BestBio, Hunan, China) according to the manufacturer’s instructions. The concentrations of Ca^2+^ in the cytoplasm and Golgi vesicles were detected using the Ca^2+^ Assay Kit (Nanjing Jiancheng Bioengineering Institute) according to the manufacturer’s protocol.

For the ipsilateral brain samples of SD rats, intracellular Ca^2+^ was measured in Fluo-3/acetoxymethyl (AM)-loaded cells by flow cytometry. Briefly, the brain tissues were digested and then incubated with 5 μM Fluo-3/AM (Beyotime) at 37°C for 0.5 h according to the instructions of the manufacturer. After washing and resuspension in PBS, intracellular Ca^2+^ levels were measured at an excitation wavelength of 488 nm and an emission wavelength of 530 nm using a flow cytometer (Beckman).

### Electron Microscope Test

An electron microscope specimen was prepared as previously described (Fan et al., 2016) and then observed with a Hitachi HT7700 transmission electron microscope (Tokyo, Japan). Section analyses were all under the same intensity condition and the same magnification of the electron microscope.

### Infarct Volume Analysis

The mice were sacrificed on day 7 after reperfusion and the brains were removed quickly. Infarct volumes were measured by 2,3,5-triphenyltetrazolium chloride (TTC) staining. All brain slices of mice from each group (*n* = 3 animals per group) were used to perform TTC staining. Slices were incubated in 2% TTC solution for 30 min at 37.0°C, then fixed in 10% formalin in the border zone of infarction, and were outlined with Image-Pro Plus Analysis Software (Media Cybernetics, Bethesda, MD, United States). The analysis was done by investigators who were blinded to the experimental groups.

### Behavioral Analysis

The modified neurologic severity score (mNSS) and rotarod treadmill were used to evaluate the neurological deficits of rats in each group before they were killed. All rats in each group received behavioral analysis on days 0 (pre-MCAO), 1, 3, and 7 after reperfusion. The mNSS consists of motor, sensory, reflex, and balance tests and was used to grade the neurological function on a scale of 0–18 (Zhao et al., 2020). For the rotarod treadmill, the rats were placed on rotating rods which accelerated at 3–20 rpm for 5 min. The time that the animal remained on the rod was the measured parameter. Two observers blinded to the treatment and grouping were assigned to conduct behavioral analysis.

### shRNA Knockdown of SPCA1

For short hairpin RNA (shRNA) knockdown, we chose the shRNA target sequence 5′-ccggccTGCGGACTTACGCTTATTT ctcgagAAATAAGCGTAAGTCCGCAggtttttg-3′. N2a cells were silenced with SPCA1 shRNA by using a shRNA transfection kit according to the manufacturer’s instruction (GIEE, China). The efficiency of the knockdown of SPCA1 in N2a cells was verified by qPCR and Western blot.

### Statistical Analysis

All statistical analyses were performed using SPSS statistical software (SPSS, Inc., Chicago, IL, United States). After testing for normal distribution, the data of two independent variables were analyzed using Mann–Whitney test. For three or more variables, Kruskal–Wallis test was performed followed by *post hoc* analysis using Tukey’s test. All data are expressed as mean ± SEM. Differences between the mean values were considered significant at *P* < 0.05.

## Results

### Characterization of OM-MSCs

**FIGURE 1:**
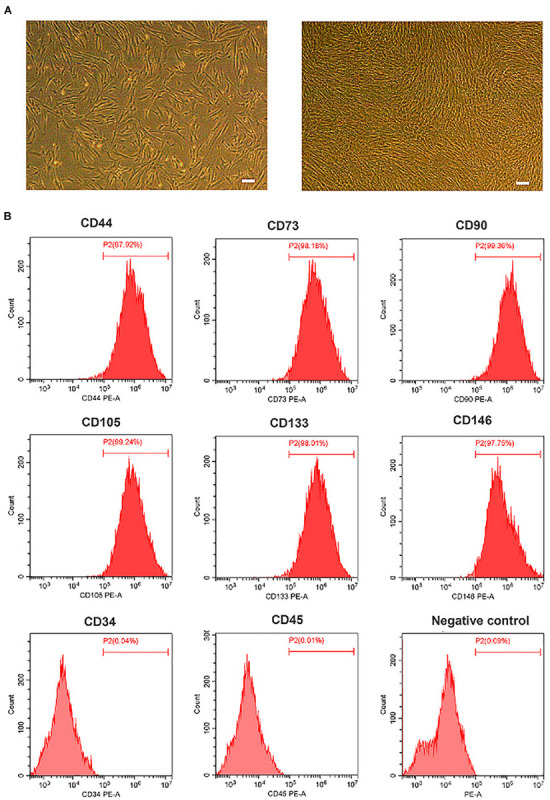
Characterization of olfactory mucosa mesenchymal stem cells (OM-MSCs). **(A)** Morphology of OM-MSCs in cell culture. Cultured cells [day 1 after passage (*left*), day 3 after passage (*right*)] showed typical fibroblastic or spindle morphology (*scale bar*, 200 μm). **(B)** Immunophenotype of OM-MSCs at passage 3 identified by flow cytometry analysis.

The morphology of OM-MSCs was typically fibroblastic or in spindle form, as shown in Figure 1A. The immunophenotype of OM-MSCs identified by flow cytometry exhibited positive expression of MSC markers (CD44, CD73, CD90, CD105, CD133, and CD146) and negative expression of hematopoietic stem cell (HSC) markers (CD34 and CD45; Figure 1B).

### OM-MSCs Ameliorated OGD/R-Induced Apoptosis and Oxidative Stress in N2a Cells

**FIGURE 2:**
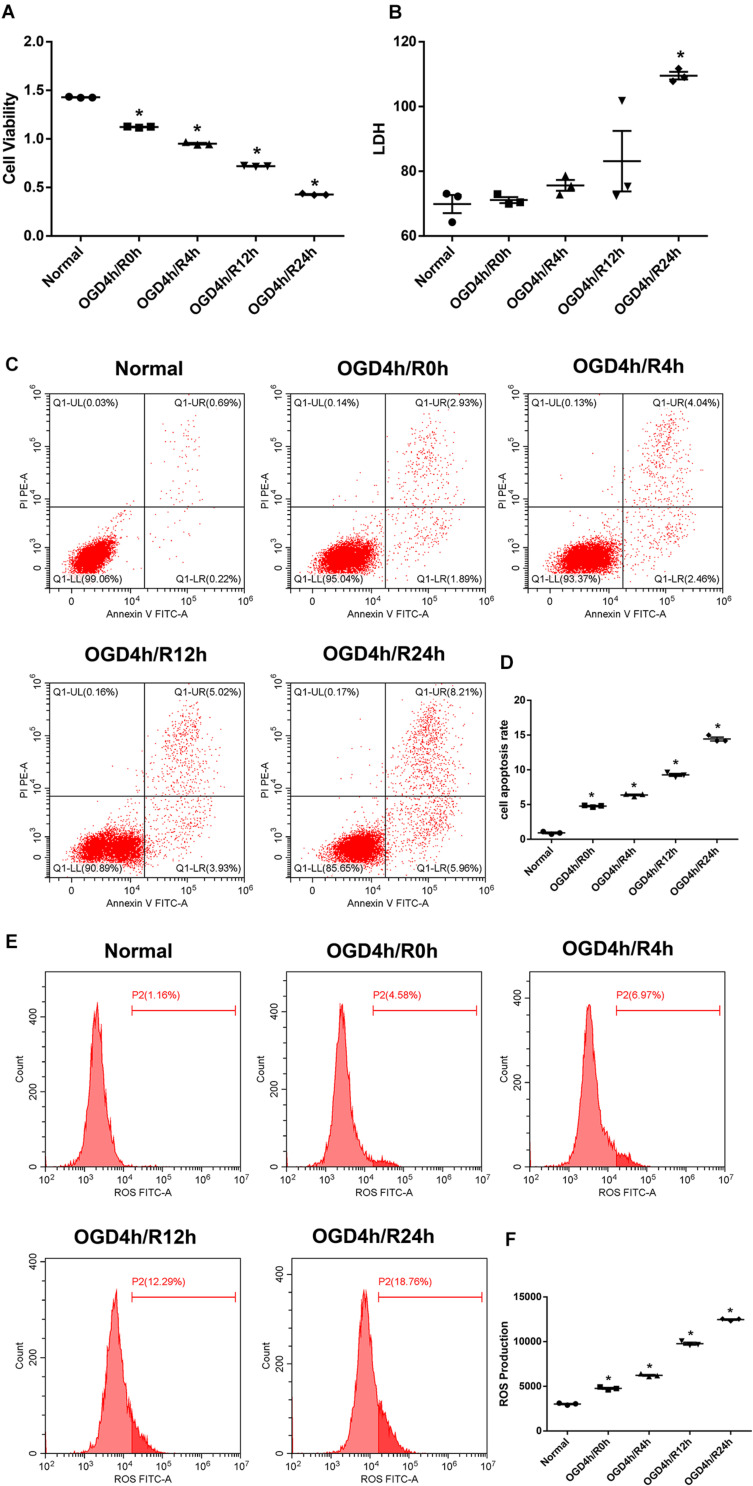
Oxygen and glucose deprivation/reoxygenation (OGD/R) induce cell injury in N2a cells. **(A)** Cell viability was determined by the Cell Counting Kit-8 (CCK-8) assay. **(B)** Cell death was determined by the lactate dehydrogenase (LDH) assay. **(C,D)** Apoptosis was evaluated by flow cytometry analysis. **(E,F)** Intracellular reactive oxygen species (ROS) was detected using an oxidation-sensitive fluorescent probe (DCFH-DA). Data are shown as the mean ± SEM based on three independent experiments. *p ≤ 0.05, compared with the normal group.

Oxygen and glucose deprivation/reoxygenation-induced N2a cell injury was performed as a classical model to mimic cerebral IRI *in vitro*. After 4 h OGD exposure, we treated N2a cells with different time courses of reoxygenation. The cell viability was significantly decreased with the time development compared with the normal group (Figure 2A), while the LDH, apoptosis rate, and ROS level were apparently increased and reached the highest changes at the 24-h reoxygenation time point (Figures 2B–F). Thereby, a 4-h OGD treatment followed by a 24-h reoxygenation therapy were applied for further experiments.

**FIGURE 3:**
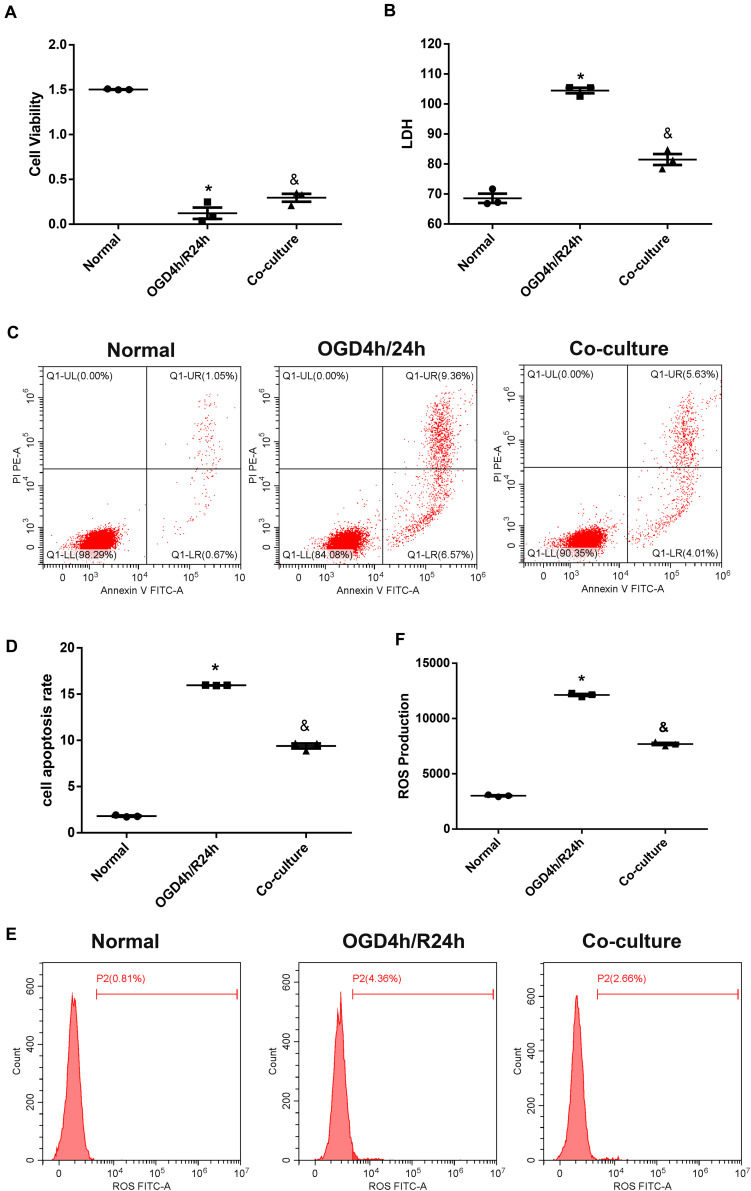
Olfactory mucosa mesenchymal stem cells (OM-MSCs) attenuated oxygen and glucose deprivation/reoxygenation (OGD/R)-induced apoptosis and oxidative stress in N2a cells. **(A)** Cell viability was determined by the Cell Counting Kit-8 (CCK-8) assay. **(B)** Cell death was determined by the lactate dehydrogenase (LDH) assay. **(C,D)** Apoptosis was evaluated by flow cytometry analysis. **(E,F)** Intracellular reactive oxygen species (ROS) was detected using an oxidation-sensitive fluorescent probe (DCFH-DA). Data are shown as the mean ± SEM based on three independent experiments. *p ≤ 0.05, compared with the normal group and ^&^*p* ≤ 0.05, compared with the OGD4h/R24h group.

We then used a Transwell device to co-culture N2a cells with OM-MSCs to investigate whether OM-MSCs could rescue N2a cells from OGD/R injury. The results are shown in Figure 3. OM-MSC co-culture notably reversed the decline in cell viability after OGD/R insult; meanwhile, the upregulated LDH production, apoptosis rate, as well as ROS level were also markedly reduced *via* OM-MSC co-culture upon OGD/R-induced injury (Figures 3A–F).

### OM-MSCs Alleviated Neuronal Apoptosis and Oxidative Stress in I/R Rats

**FIGURE 4:**
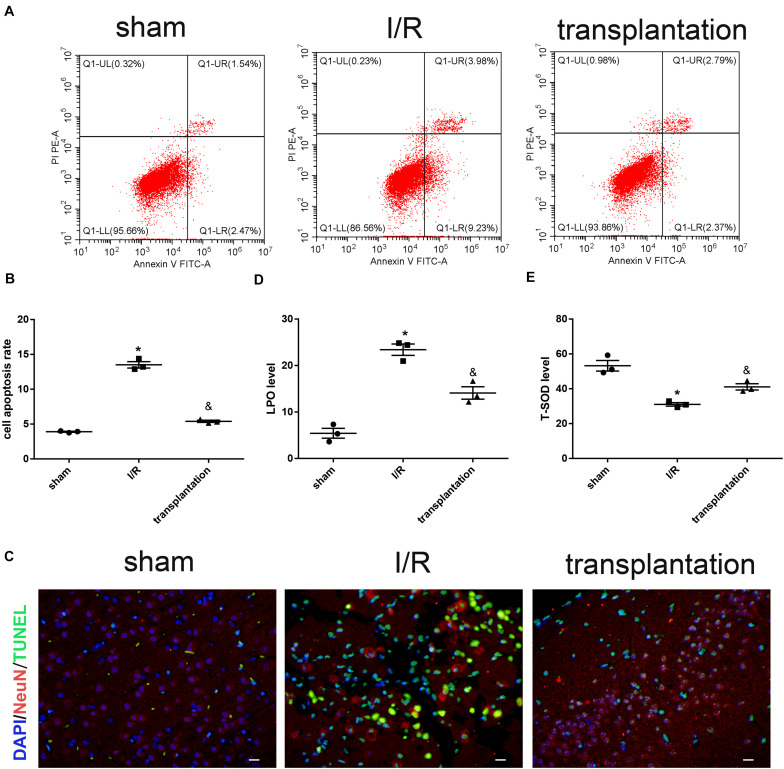
Olfactory mucosa mesenchymal stem cells (OM-MSCs) alleviated neuronal apoptosis and oxidative stress in ischemia/reperfusion (I/R) rats. **(A,B)** Apoptosis of ipsilateral brain samples was evaluated by flow cytometry analysis. **(C)** Representative images of NeuN and TUNEL double immunostaining (*scale bar*, 40 μm). **(D,E)** Lipid peroxidation (LPO) and total superoxide dismutase (T-SOD) levels of ipsilateral brain samples were measured by the LPO assay and T-SOD assay kits, respectively. Data are shown as the mean ± SEM (*n* = 3 animals per group). *p ≤ 0.05, compared with the sham group and ^&^*p* ≤ 0.05, compared with the I/R group.

We then established the MCAO rat model to achieve cerebral IRI *in vivo*. The apoptosis rate in the I/R group was significantly higher than that in the sham group. NeuN and TUNEL double immunostaining further verified the increased neuronal apoptosis in I/R rats. Meanwhile, neuronal apoptosis induced by cerebral ischemia/reperfusion was notably alleviated by OM-MSC injection in the transplantation group rats (Figures 4A–C).

We also examined the oxidative stress level in each group of rats. Compared with the sham group, the LPO level was elevated while the T-SOD level was reduced in the I/R group. The opposite alterations of these two indicators supported that cerebral ischemia/reperfusion could indeed lead to increased levels of oxidative stress. Likewise, OM-MSC injection could downregulate the LPO level and upregulate the T-SOD level in I/R rats (Figures 4D,E).

### OM-MSCs Reduced Cerebral Infarction Volume and Improved Neurologic Deficits in I/R Rats

**FIGURE 5:**
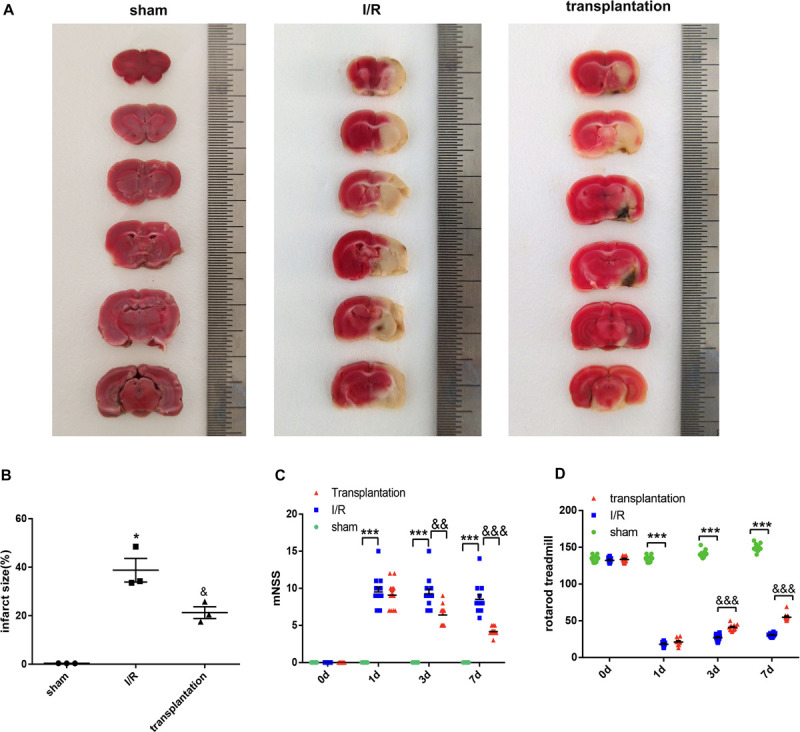
Olfactory mucosa mesenchymal stem cell (OM-MSC) injection reduced the cerebral infarction volume and alleviated neurologic deficits in ischemia/reperfusion (I/R) rats. **(A,B)** The infarct volume was determined by 2,3,5-triphenyltetrazolium chloride (TTC) staining (*n* = 3 animals per group). **(C,D)** The *line charts* show the results of the modified neurologic severity score (mNSS; *n* = 12 animals per group) and rotarod treadmill (*n* = 12 animals per group). Data are shown as the mean ± SEM. *p ≤ 0.05, ***p < 0.001, compared with the sham group and ^&^*p* ≤ 0.05, ^&&^*p* < 0.01, and ^&&&^*p* < 0.001, compared with the I/R group.

The cerebral infarction volume in each group was examined by TTC staining. No infarction was observed in the sham group, while a white infarct lesion occurred in the I/R and transplantation groups, suggesting successful establishment of the MCAO model in rats. The infarction size in the transplantation group was significantly diminished, indicating that OM-MSC injection was able to reduce the cerebral infarction volume (Figures 5A,B).

Behavioral function was evaluated by the mNSS and rotarod treadmill. The mNSS was remarkably increased in the I/R 1-day, I/R 3-day, and I/R 7-day groups compared with that of the sham group and notably decreased in the transplantation 3-day and transplantation 7-day groups compared with that in the I/R group (Figure 5C). The rotarod treadmill results were also obviously improved by OM-MSC transplantation at 3 and 7 days (Figure 5D). In brief, our results confirmed that OM-MSC injection could improve neurologic deficits in I/R rats.

### OM-MSCs Upregulated SPCA1 Expression in OGD/R-Treated N2a Cells and I/R Rats

**FIGURE 6:**
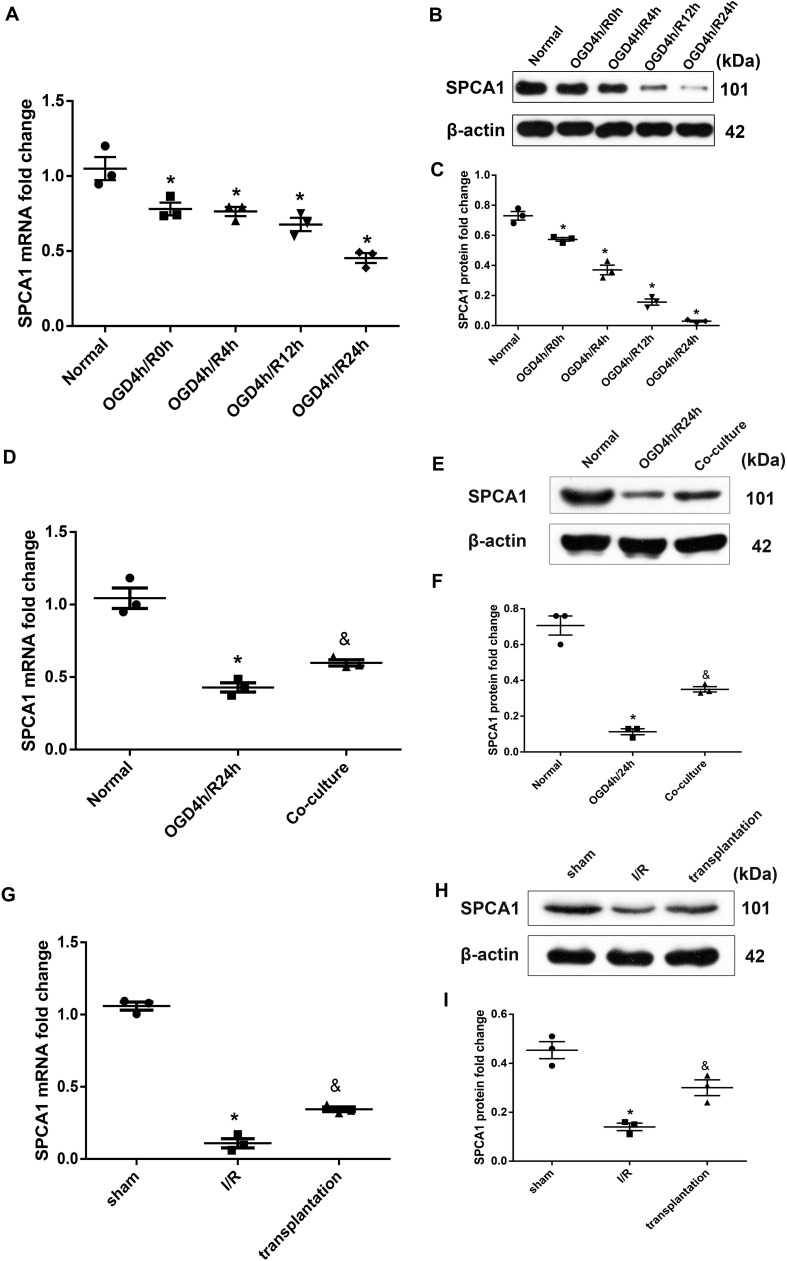
Olfactory mucosa mesenchymal stem cells (OM-MSCs) upregulated SPCA1 expression in oxygen and glucose deprivation/reoxygenation (OGD/R)-treated N2a cells and ischemia/reperfusion (I/R) rats. **(A–C)** SPCA1 messenger RNA (mRNA) and protein expressions of OGD/R-treated N2a cells at different time points were visualized by quantitative PCR (qPCR) and Western blot. **(D–F)** SPCA1 mRNA and protein expressions of N2a cells in the normal, OGD4h/R24h, and OM-MSC co-culture groups were measured by qPCR and Western blot. **(G–I)** SPCA1 mRNA and protein expressions of rats’ ipsilateral brain samples in the sham, I/R, and OM-MSC transplantation groups were detected by qPCR and Western blot. Data are shown as the mean ± SEM based on three independent experiments. *p ≤ 0.05, compared with the normal or sham group and ^&^*p* ≤ 0.05, compared with the OGD4h/R24h or I/R group.

The expression level of SPCA1 in N2a cells was identified to be decreased after OGD/R insult compared to the normal group, the alteration reaching a maximum in the 24-h reoxygenation at both the mRNA and protein levels (Figures 6A–C). OM-MSCs were able to upregulate the expression of SPCA1 in N2a cells after OGD/R injury (Figures 6D–F).

Similarly, cerebral ischemia/reperfusion contributed to a remarkable decline in the mRNA and protein levels of SPCA1 in the I/R group compared with the sham group, and OM-MSC transplantation was capable of upregulating the SPCA1 protein expression in I/R rats (Figures 6G–I).

### OM-MSCs Attenuated Ca^2+^ Overload and Improved GA Morphology in OGD/R-Treated N2a Cells and I/R Rats

**FIGURE 7:**
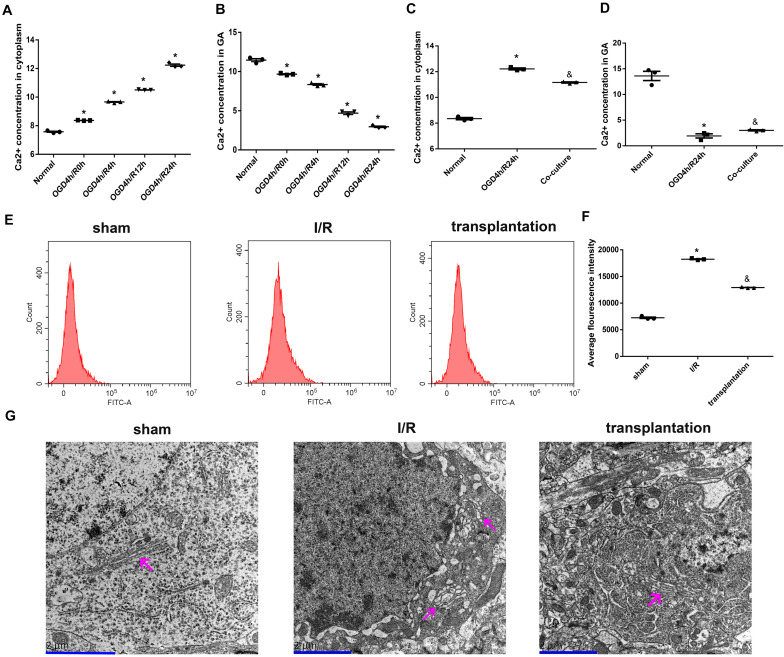
Olfactory mucosa mesenchymal stem cells (OM-MSCs) attenuated Ca^2+^ overload and improved Golgi apparatus (GA) morphology in oxygen and glucose deprivation/reoxygenation (OGD/R)-treated N2a cells and ischemia/reperfusion (I/R) rats. **(A,B)** Ca^2+^ concentrations in the cytoplasm and GA of OGD/R-treated N2a cells at different time points were determined by the Ca^2+^ Assay Kit. **(C,D)** Ca^2+^ concentrations in the cytoplasm and GA of N2a cells in the normal, OGD4h/R24h, and OM-MSC co-culture groups were measured by the Ca^2+^ Assay Kit. **(E,F)** Intracellular Ca^2+^ of rats’ ipsilateral brain samples in the sham, I/R, and OM-MSC transplantation groups were detected by flow cytometry analysis using a Fluo-3/AM kit. **(G)** Representative image of GA ultramicrostructure changes by using an electron microscope (*scale bar*, 2 μm). The GA was indicated by the *magenta arrow*. Data are shown as the mean ± SEM based on three independent experiments. *p ≤ 0.05, compared with the normal or sham group and ^&^*p* ≤ 0.05, compared with the OGD4h/R24h or I/R group.

Due to the function of SPCA1 being associated with intracellular Ca^2+^ homeostasis, we subsequently measured the intracellular Ca^2+^ concentrations and discovered a notably increased Ca^2+^ concentration in the cytoplasm after OGD/R exposure while a decreased Ca^2+^ concentration in GA, both of which reached the highest changes in the 24-h time point (Figures 7A,B). Interestingly, after OM-MSC co-culture following OGD/R insult, the increased Ca^2+^ concentration in the cytoplasm was obviously alleviated (Figure 7C), while the Ca^2+^ concentration in GA was upregulated (Figure 7D).

We also assayed the intracellular Ca^2+^ concentration in the rats from each group and observed an elevated Ca^2+^ concentration in I/R rats; likewise, OM-MSC transplantation could notably repress the increase of Ca^2+^ concentration in I/R rats (Figures 7E,F).

Moreover, we examined the GA ultramicrostructure changes of neurons using an electron microscope. As visualized in Figure 7G, neurons in the sham group had GA with normal morphology and structure, accompanied by the endoplasmic reticulum, lysosomes, mitochondria, nerve microfilaments, neural tubes, and a complete double nuclear membrane. In the I/R group, the GA was swollen and dissolved, other organelles were also fractured, and the nuclear membrane became blurred. However, the GA was less edematous in the transplantation group. Collectively, the ultramicropathological changes of the GA in the transplantation groups were less significant compared with those in the I/R group.

### OM-MSCs Protected N2a Cells From OGD/R-Induced Injury Through Modulating SPCA1

**FIGURE 8:**
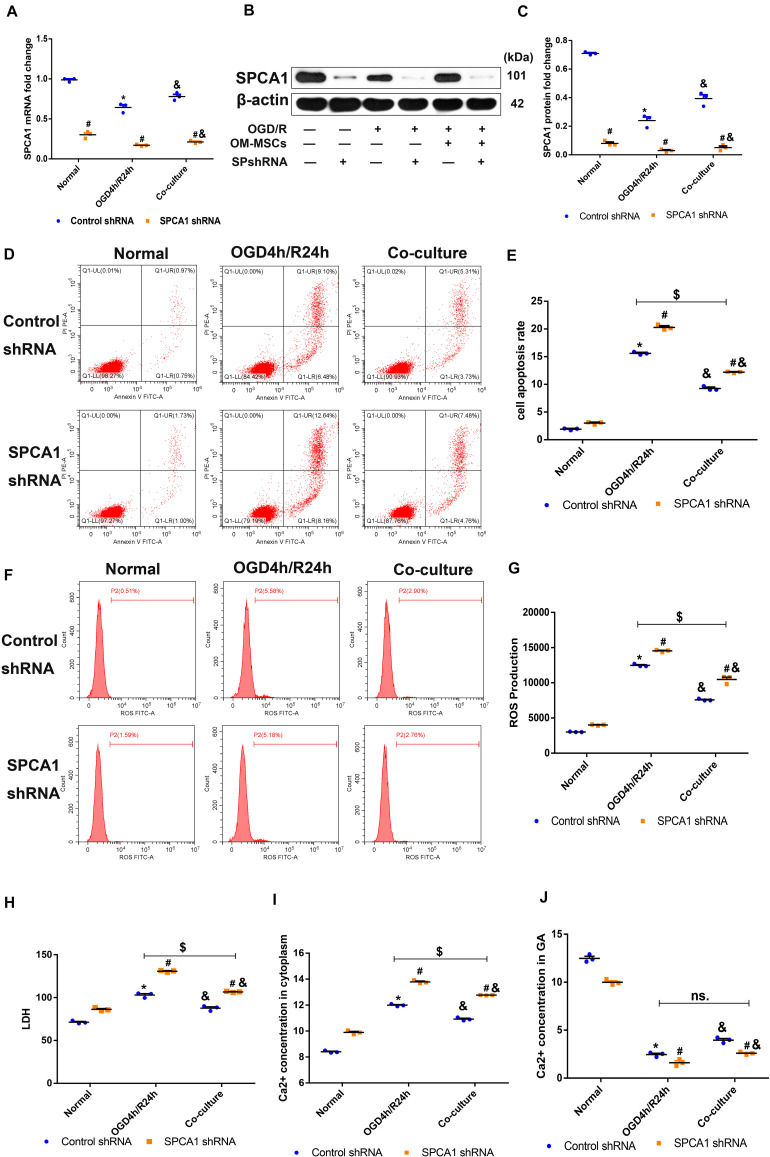
Olfactory mucosa mesenchymal stem cells (OM-MSCs) protected N2a cells from oxygen and glucose deprivation/reoxygenation (OGD/R)-induced injury through modulating SPCA1. **(A–C)** SPCA1 messenger RNA (mRNA) and protein expressions in both the control short hairpin RNA (shRNA) group and the SPCA1 shRNA group were visualized by quantitative PCR (qPCR) and Western blot. **(D,E)** Apoptosis in both the control shRNA group and the SPCA1 shRNA group was evaluated by flow cytometry analysis. **(F,G)** Intracellular reactive oxygen species (ROS) in both the control shRNA group and the SPCA1 shRNA group was detected using an oxidation-sensitive fluorescent probe (DCFH-DA). **(H)** Cell death in both the control shRNA group and the SPCA1 shRNA group was determined by the lactate dehydrogenase (LDH) assay. **(I,J)** Ca^2+^ concentrations in the cytoplasm **(I)** and the Golgi apparatus (GA; **J)** in both the control shRNA group and the SPCA1 shRNA group were determined by the Ca^2+^ Assay Kit. Data are shown as the mean ± SD based on three independent experiments. *p ≤ 0.05, compared with the normal group; ^&^*p* ≤ 0.05, compared with the OGD4h/R24h group; and ^#^*p* ≤ 0.05, compared with the control shRNA group. ^$^*p* ≤ 0.05, ns. *p* > 0.05, compared with OGD4h/R24h of the control shRNA group.

To explore the role of SPCA1 in the neuroprotective effect of OM-MSCs against cerebral IRI, plasmid containing SPCA1 shRNA sequence was constructed and transfected into N2a cells before the experiment. The transduction results were verified by PCR as well as Western blot analysis, which are shown in Figures 8A–C. Transfection with SPCA1 shRNA contributed to a notable decrease in the mRNA and protein levels compared with control shRNA.

Compared with the control shRNA group, the apoptosis rate, ROS levels, LDH production, as well as the Ca^2+^ concentration in the cytoplasm of N2a cells induced by OGD/R insult were apparently increased in the SPCA1 shRNA group, and SPCA1 depletion in N2a cells mitigated the protective effects of OM-MSCs following OGD/R injury (Figures 8D–I). Meanwhile, after OGD/R injury, the Ca^2+^ concentration in the GA of N2a cells was significantly lower in the SPCA1 shRNA group than in the control shRNA group, and SPCA1 knockdown in N2a cells restricted the capacity of OM-MSCs to upregulate the Ca^2+^ concentration in the GA of N2a cells after OGD/R insult (Figure 8J). Overall, the above findings showed that OM-MSCs protected N2a cells from OGD/R-induced injury probably through modulating SPCA1.

## Discussion

We successfully established *in vivo* and *in vitro* models of cerebral IRI, as previously described (Longa et al., 1989; Huang and Hu, 2018). It is well known that OGD/R-induced cell injury is mainly characterized by a decreased cell viability and increased apoptosis rate and LDH release level (Huang and Hu, 2018; Ma et al., 2020), and our results were consistent with previous studies. Meanwhile, significant cerebral infarction lesions were observed by TTC staining of the rats’ brain tissues, suggesting the establishment of a successful *in vivo* model.

Oxidative stress, induced by ROS during cerebral ischemia and especially reperfusion, is important in the pathological process of ischemic stroke and is critical for the cascade development of cerebral IRI (Wu et al., 2020). It results in LPO, apoptosis, and, ultimately, neuronal death together with other pathophysiological mechanisms. We also found that the ROS and LPO levels were increased while the SOD levels were decreased in our models of cerebral IRI.

Mesenchymal stem cells have exhibited therapeutic properties on IRI because of their paracrine activity, cell–cell interaction, anti-inflammatory activity, and immunomodulatory effects (Souidi et al., 2013; Barzegar et al., 2019; Oliva, 2019; Tobin et al., 2020). Leu et al. (2010) have demonstrated that intravenous injection of AD-MSCs significantly attenuated oxidative stress in an experimental ischemic stroke model. Calio et al. (2014) have concluded that the transplantation of BM-MSCs decreases oxidative stress and apoptosis in the brain of stroke rats. Alhazzani et al. (2018) have found that MSC co-culture could protect Ca^2+^ and oxidant-mediated damage in SH-SY5Y-differentiated neuronal cells. Similarly, our results also showed that OM-MSCs were able to downregulate ROS as well as LPO levels and upregulate antioxidase SOD levels in the cerebral IRI models, eventually reducing neuronal apoptosis and infarction volume. Consequently, we fully believe that OM-MSCs could also confer cerebral protection against IRI by suppressing oxidative stress.

In recent years, with the concept of “GA stress” proposed, the complex role of GA in oxidative stress has been gradually recognized (Jiang et al., 2011; Li et al., 2016). Based on the findings of Jiang et al. (2011) and He and Hu (2012), as one of the Ca^2+^ transporters of GA, SPCA1 played an important role in the process of GA maintaining intracellular Ca^2+^ homeostasis under physiological conditions. However, the activity and expression of SPCA1 were decreased during cerebral ischemia/reperfusion, and its ability to uptake intracellular Ca^2+^ was also impaired, leading to intracellular Ca^2+^ overload (Pavlíková et al., 2009; Li et al., 2015; Fan et al., 2016). It is well known that Ca^2+^ overload is another fatal molecular event in cerebral IRI (Kalogeris et al., 2016; Radak et al., 2017). Sustained excessive intracellular calcium levels often cause neuronal cell hypercontracture, proteolysis, and, eventually, death (Pittas et al., 2019). During reperfusion, the metabolites produced by oxidative stress destroy the integrity of the cell membrane and organelle membrane, and Ca^2+^ release channels on the cell membranes and organelle membranes are opened, resulting in Ca^2+^ influx into the cytoplasm from the extracellular environment and the endoplasmic reticulum or sarcoplasmic reticulum (Li et al., 2015). And more importantly, Ca^2+^ overload could also enhance oxidative stress, their interaction promoting the pathological process of an IRI cascade (Jiang et al., 2011). In this paper, we discovered a decrease in SPCA1 expression, an increase in cytoplasmic Ca^2+^ levels, and a decrease in GA Ca^2+^ levels in the ischemic stroke model, which were in line with previous studies.

Besides, the GA fragment, another typical manifestation of GA stress in ischemic stroke, was often induced by oxidative stress and apoptosis (Zhong et al., 2015; Zhang et al., 2019). The damage of microtubule proteins mainly contributed to the fragmentation and even dissolution of the GA during oxidative stress and apoptosis. Our results presented that the GA was swollen and dissolved in the neuron of I/R rats, which was in accordance with existing evidences.

Previous researches on the neuroprotective role of MSCs at the subcellular organelle level in ischemic stroke always focused on the mitochondria and endoplasmic reticulum (Xing et al., 2016; Mahrouf-Yorgov et al., 2017). The impact of stem cell therapy on the function and morphology of GA after cerebral IRI was uncovered. Based on the results above, our results firstly demonstrated that OM-MSCs were able to upregulate SPCA1 expression, rescue its function of maintaining intra-Golgi Ca^2+^ homeostasis, and reduce the edema and dissolution of GA in neurons of ischemic stroke models.

Since SPCA1 has previously been shown to exhibit anti-oxidative stress and anti-apoptotic effects in ischemic stroke (Uccelletti et al., 2005; Sepulveda et al., 2009), the upregulation of SPCA1 expression and other neuroprotective effects of OM-MSCs in an ischemia/reperfusion model have also been confirmed according to our results. As a result, we speculated whether the neuroprotective effect of OM-MSCs on cerebral IRI was associated with its ability to upregulate SPCA1 expression and rescue its function in neurons. Subsequently, we used SPCA1 shRNA to construct a SPCA1 knockout model in N2a cells and found that SPCA1 shRNA partly restricted the capacity of OM-MSCs to alleviate OGD/R-induced apoptosis and elevated the ROS levels and LDH production as well as the intracellular Ca^2+^ overload. The above findings suggested that the expression and function of SPCA1 in neurons were relevant to the neuroprotective effect of OM-MSCs on cerebral IRI.

Nevertheless, we also observed that OM-MSCs still had a partial protective effect, including reduced apoptosis and ROS production and regulated Ca^2+^ concentration in the cytoplasm, on OGD/R-induced cell injury in the case of SPCA1 being knocked down. We considered that this outcome was related to the function diversity and plasticity of OM-MSCs. Previous studies have concluded that MSCs exhibited anti-oxidative, anti-apoptotic, endogenous neurogenesis, synaptogenesis, angiogenesis, anti-inflammatory, and immunomodulatory effects in ischemic stroke (Souidi et al., 2013; Hao et al., 2014), and MSCs exerted their neuroprotective effects partly by secreting neurotrophic factors, such as VEGF, NGF, BDNF, and bFGF, etc. (Stonesifer et al., 2017; Barzegar et al., 2019). The secretome of OM-MSCs has been identified by Ge et al. (2016), and their results showed that these secreted proteins were associated with neurotrophy, angiogenesis, cell growth, differentiation, and apoptosis. Consequently, OM-MSCs may be capable of attenuating cerebral IRI partly by secreting a series of neurotrophic factors. Those molecules may reduce apoptosis and oxidative stress levels by acting on the mitochondria and endoplasmic reticulum when SPCA1 was blocked. That the function of the injured mitochondria and endoplasmic reticulum could be rescued by other types of MSCs has been confirmed by previous researchers (Chi et al., 2018; Tseng et al., 2020). In terms of the regulation of intracellular Ca^2+^ by OM-MSCs during cerebral ischemia/reperfusion when SPCA1 was knocked down, the possible mechanisms were as follows: firstly, OM-MSCs reduced the oxidative stress level through other feasible pathways, which inhibited the Ca^2+^ influx from extracellular stores and the endoplasmic reticulum, eventually leading to a decline in the Ca^2+^ concentration of the cytoplasm. Secondly, in addition to GA, there were also Ca^2+^ uptake channels in the endoplasmic reticulum membrane, which were also impaired by IRI (Jiang et al., 2011). OM-MSCs may be able to rescue these channels and subsequently reduce the Ca^2+^ overload in the cytoplasm to a certain extent. Accordingly, OM-MSCs could still exhibit part of the ability to reduce apoptosis as well as ROS production and regulate the Ca^2+^ concentration after SPCA1 knockdown.

Nowadays, cell-based therapies are considered to be one of the most promising options to radically advance ischemic stroke treatment (Boltze et al., 2019), and animal models of ischemic stroke are indispensable for their translation into clinical trials. Hence, the establishment of a highly efficient and predictable animal model is conductive to improve the quality of preclinical researches regarding cell therapy (Kringe et al., 2020). In the present study, the choice of male rats effectively eliminated the influence of female sex hormones on the effect of cell therapy, and the standardization of the animal housing conditions greatly reduced its impact on the neurological endpoint. Randomized grouping and allocation concealment also avoided the limitations of other confounding factors to some degree (Boltze et al., 2017; Bosetti et al., 2017). Additionally, the reperfusion model we used here was in line with the recommendations of the guidelines for the study of neuroprotective therapies in recanalization scenarios (Savitz et al., 2019). Consequently, we believed that these advantages would make our results more credible.

However, there are still some limitations regarding this study, which are expected to be improved on in subsequent studies. The first is the small sample size. It would be significant to perform the examination of this treatment in a large cohort for subsequent confirmation. Secondly, the mNSS and rotarod treadmill are widely used neurofunctional assessments in experimental stroke, but other evidences indicated that the mNSS and rotarod are not perfectly fit for neurofunctional assessments after stroke in the context of MSC-based therapies since these two behavioral tests could not distinguish recovery from compensatory behavior well (Boltze et al., 2014; Balkaya et al., 2018). Therefore, it is recommended to choose behavioral tests that are minimally affected by behavior compensation in future experimental stroke, such as Montoya’s staircase and the cylinder test. Thirdly, studies on other types of cells have found that cryopreservation limited the effectiveness of those cell types (Weise et al., 2014). A similar exploration should also be carried out in cryopreserved OM-MSCs. Fourthly, no immunosuppressive agent was used, although in xenotransplantation, the possible graft rejection could directly influence the therapeutic outcome of OM-MSCs. However, other investigators recommended MSCs as a novel immunomodulatory strategy in preclinical transplantation studies due to their immunosuppressive properties (Diehl et al., 2017), suggesting that MSCs could be applied relatively safely in non-autologous approaches. Lastly, the establishment of the SPCA1 gene knockout rats is promising. It will provide a more profound understanding of the mechanism regarding SPCA1 in the neuroprotective effect of OM-MSCs on cerebral IRI.

In summary, our findings suggest that OM-MSCs may be a useful candidate of cell therapies for the treatment of ischemic stroke. OM-MSCs exert neuroprotective effects against cerebral IRI, probably *via* modulating SPCA1 and reducing the edema and dissolution of the GA in neurons. Further studies will be conducted to highlight the role of SPCA1 in the neuroprotection of OM-MSCs *in vivo* by constructing gene knockout animal models of ischemic stroke.

## Data Availability Statement

The raw data supporting the conclusions of this article will be made available by the authors, without undue reservation.

## Ethics Statement

The studies involving human participants were reviewed and approved by Ethical Committee of Hunan Normal University. The patients/participants provided their written informed consent to participate in this study. The animal study was reviewed and approved by Laboratory Animal Ethics Committee of the Second Affiliated Hospital of Hunan Normal University.

## Author Contributions

ZH acquired the funding. JH attended in research design, experimental performances except animal behavioral tests, data analysis, and drafting the manuscript. JL participated in cell culture and animal behavioral tests. YH participate in cell culture and data analysis. YZ participated in animal experiment and behavioral tests. WC took part in animal experiment. DD and XT discussed the results. ZH and ML took care of all aspects including research design, data analysis, and manuscript preparation. All authors read and approved the final manuscript.

## Conflict of Interest

The authors declare that the research was conducted in the absence of any commercial or financial relationships that could be construed as a potential conflict of interest.

## Abbreviations

*CCK-8*: Cell Counting Kit-8
*GA*: Golgi apparatus
*HSC*: hematopoietic stem cell
*iPSCs*: induced pluripotent stem cells
*I/R*: ischemia/reperfusion
*IRI*: ischemia/reperfusion injury
*LDH*: lactate dehydrogenase
*LPO*: lipid peroxidation
*MCAO*: middle cerebral artery occlusion
*mNSS*: modified neurologic severity score
*MSCs*: mesenchymal stem cells
*NSC*: neural stem cells
*OGD/R*: oxygen and glucose deprivation/reoxygenation
*OM-MSCs*: olfactory mucosa mesenchymal stem cells
*ROS*: reactive oxygen species
*SPCA*: secretory pathway Ca ^2 +^ -ATPase
*SPCA1*: secretory pathway Ca ^2 +^ -ATPase isoform1
*T-SOD*: total superoxide dismutase.
